# Pancreatic cancer mortality in Serbia from 1991-2010 – a joinpoint analysis

**DOI:** 10.3325/cmj.2013.54.369

**Published:** 2013-08

**Authors:** Milena Ilić, Hristina Vlajinac, Jelena Marinković, Nikola Kocev

**Affiliations:** 1 Department of Epidemiology, Faculty of Medical Sciences, University of Kragujevac, Kragujevac, Serbia; 2 Institute of Epidemiology, Faculty of Medicine, University of Belgrade, Belgrade, Serbia; 3 Institute of Medical Statistics and Informatics, Faculty of Medicine, University of Belgrade, Belgrade, Serbia

## Abstract

**Aim:**

To analyze the trends of pancreatic cancer mortality in Serbia.

**Methods:**

The study covered the population of Serbia in the period 1991 to 2010. Mortality trends were assessed by the joinpoint regression analysis by age and sex.

**Results:**

Age-standardized mortality rates ranged from 5.93 to 8.57 per 100 000 in men and from 3.51 to 5.79 per 100 000 in women. Pancreatic cancer mortality in all age groups was higher among men than among women. It was continuously increasing since 1991 by 1.6% (95% confidence interval [CI] 1.1 to 2.0) yearly in men and by 2.2% (95% CI 1.7 to 2.7) yearly in women. Changes in mortality were not significant in younger age groups for both sexes. In older men (≥55 years), mortality was increasing, although in age groups 70-74 and 80-84 the increase was not significant. In 65-69 years old men, the increase in mortality was significant only in the period 2004 to 2010. In ≥50 years old women, mortality significantly increased from 1991 onward. In 75-79 years old women, a non-significant decrease in the period 1991 to 2000 was followed by a significant increase from 2000 to 2010.

**Conclusion:**

Serbia is one of the countries with the highest pancreatic cancer mortality in the world, with increasing mortality trend in both sexes and in most age groups.

According to the GLOBOCAN 2008 estimates, pancreatic cancer causes more than 270 000 deaths per year (accounts for 3.5% of all deaths), ranking ninth among the leading causes of cancer death in both sexes together (1-3). It is one of the most lethal malignant neoplasms, with the 5-year survival rate of less than 5% (4,5).

The majority of pancreas cancer deaths (61%) occurs in developed countries, where pancreatic cancer is the fifth leading cause of death in men and the fourth in women (1,3,4). The highest mortality rates of pancreatic cancer are reported in North America (6.9 per 100 000) and Europe (from 6.6 per 100 000 in western Europe to 5.9 in southern Europe) and the lowest in less developed countries in South Asia and Central Africa (approximately 1.5 per 100 000) (3).

Pancreatic cancer is more common in men than women – in 2008, age-adjusted worldwide mortality rates were 4.2 per 100 000 in men and 3.1 per 100 000 in women (3,4). It is predominantly a disease of the elderly, and almost 90% of all cases are diagnosed after the age of 55 years (3). In Serbia in 2008, pancreatic cancer was the fifth most common cause of cancer mortality in both sexes, with death rates similar to those in developed countries (8.3 per 100 000 in men and 5.5 in women) (3).

In the recent decade, pancreatic cancer mortality in developed countries has been increasing (for example, in the USA by 0.5% per year for both sexes; in Japan by 0.6%, but only in women; in the European Union, also only in women) (6-8). Since there are no corresponding data for the Serbian population, the aim of the present study was to analyze time trends of pancreatic cancer mortality in Serbia for the period 1991-2010.

## MATERIAL AND METHODS

### Data sources

Data on individuals who died of pancreatic cancer (code 157 revision 9 of the International Classification of Diseases from 1991 to 1996, and code C25 revision 10 from 1997) (9,10) were obtained from the Statistical Office of the Republic of Serbia (unpublished data). All deaths occurring in Serbia are registered in death files (certificate of death and a special statistical form – DEM 2) (11). A death certificate is obtained from an authorized physician in a health care organization, a coroner, or a forensic physician. Death files are checked by the local registrar and forwarded to the referral public health institute, where they are checked again and if necessary corrected by another trained medical doctor or specialist. The research included the entire population of Serbia in the period 1991 to 2010, excluding the Autonomous Province of Kosovo and Metohia, for which data have been unavailable since 1998. Data on the number and distribution according to sex and age were obtained from the population censuses in 1991 and 2002 (12). For inter-census years, the estimates published by the Statistical Office of the Republic of Serbia were used (12). Data for internally displaced persons and refugees were included in the population of Serbia and could not have been regarded as a special contingent.

### Statistical analysis

Three types of death rates were calculated: crude, age-specific, and age-standardized calculated by the direct method from the world standard population (13) and expressed as deaths per 100 000 persons stratified by sex. Age-specific rates were computed for 5-year age groups. Rates are not presented for the age subgroups (<40 years) with fewer than five pancreatic cancer deaths in any year in each of the quinquenniums.

Pancreatic cancer mortality trends were assessed by joinpoint regression analysis (Joinpoint Regression software, version 3.5.3, available through the Surveillance Research Program of the US National Cancer Institute). The significance level was set at *P* ≤ 0.05. Each joinpoint marks a significant change in the trend, and an annual percentage of change (APC) and the corresponding 95% confidence interval are computed from each trend using generalized linear models assuming a Poisson distribution (14). In the final model, the joinpoint analysis also provides an average annual percentage change (AAPC) – a summary measure over a fixed interval, which is computed as a weighted average of the APCs from the joinpoint model. We used age-standardized rate as the dependent variable and year as the independent variable. The by-variables were sex and age group. A number of joinpoints was set between 0 and 4. Grid Search method was selected (15). The minimum number of data points from the beginning of the series was set at 5 and there were at least 4 data points between two joinpoints. The permutation test was used to select the best joinpoint models with overall significance level of 0.05 and 4499 randomly selected data sets (16). We used a specific procedure – comparability test to compare two segmented line regression functions. All two-way combinations of the sex and age groups by variable were tested. The main goal of the comparability test was to compare two sets of trend data whose mean functions are represented by joinpoint regression. Specific interests were to test (i) whether two joinpoint regression functions were identical (test of coincidence) or (ii) whether two regression mean functions were parallel (test of parallelism) (17).

## RESULTS

In Serbia from 1991 to 2010, more than 16 000 inhabitants died from pancreatic cancer (approximately 9000 men and 7000 women) (Table 1). Age-standardized mortality rates ranged from 5.93 to 8.57 per 100 000 in men and from 3.51 to 5.79 per 100 000 in women. In both sexes the highest rates were recorded in 2010.

**Table 1:** Pancreatic cancer mortality in Serbia, excluding the Autonomous Province of Kosovo and Metohia, in the period 1991-2010, by sex. Number of cases, crude rate, and age standardized rate (ASR, per 100 000, using world standard population)

	Men			Women		
Year	N	crude rate	ASR	N	crude rate	ASR
1991	392	10.55	7.03	246	6.34	3.50
1992	366	9.85	6.41	265	6.82	3.76
1993	335	9.00	5.93	261	6.70	3.51
1994	356	9.56	6.17	273	7.00	3.65
1995	354	9.50	6.01	307	7.87	4.10
1996	412	11.06	6.87	296	7.59	3.80
1997	396	10.68	6.64	343	8.82	4.42
1998	416	11.28	6.77	337	8.68	4.28
1999	438	11.94	7.11	344	8.86	4.28
2000	401	10.97	6.48	346	8.96	4.28
2001	445	12.20	7.04	327	8.48	4.00
2002	466	12.78	7.22	369	9.58	4.49
2003	451	12.40	6.98	404	10.51	4.88
2004	476	13.12	7.32	376	9.78	4.29
2005	518	14.32	8.07	470	12.29	5.31
2006	520	14.43	7.99	390	10.24	4.54
2007	491	13.68	7.42	475	12.52	5.13
2008	561	15.70	8.29	473	12.53	5.17
2009	546	15.34	8.19	474	12.60	5.01
2010	566	15.96	8.57	522	13.94	5.79

**Figure 1:**
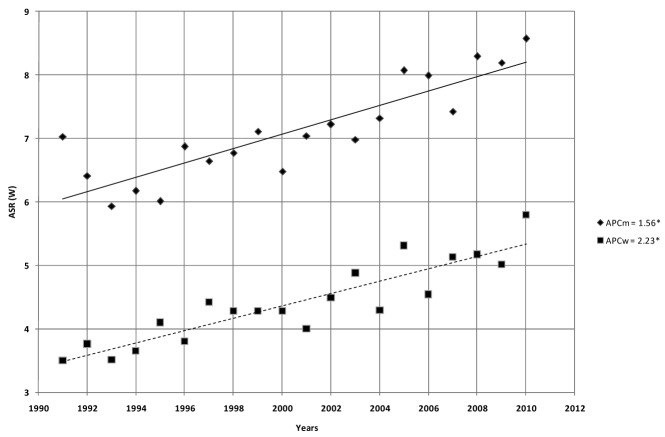
Joinpoint regression analysis of pancreatic cancer mortality in Serbia, excluding the Autonomous Province of Kosovo and Metohia, in the period 1991-2010, by sex. ASR (**W**) – age standardized rate (per 100 000, using world standard population); APC – annual percent change. *The APC change is significant (two-sided *P* < 0.05).

Pancreatic cancer mortality was continuously increasing since 1991 by 1.6% (95% confidence interval [CI] 1.1 to 2.0) yearly in men and by 2.2% (95% CI 1.7 to 2.7) yearly in women (Figure 1, Table 1). According to comparability test, pancreatic mortality trends in men and women were parallel (final selected model failed to reject parallelism, *P* = 0.243).

Mortality from pancreatic cancer was higher among men than among women in all age groups (Table 2). There were some sex differences in time trends by age. Among men, changes in mortality were small and not significant in younger age groups (<55 years), but in participants 55 years and older mortality was increasing, although in the age groups 70-74 and 80-84 the increase was not significant. In 65-69 years old men, the increase in mortality was significant only in the period from 2004 to 2010. Among women, changes in mortality in younger age groups (40-44 and 45-49 years) were not significant, but in older age groups mortality significantly increased from 1991 onward. The exception were 75-79 years old women, in whom a non-significant decreasing trend in the period 1991 to 2000 was followed by a significant increase from 2000 to 2010. According to comparability test, mortality trend in 50-54 years old men and 75-79 and 85+ years old women differed significantly from mortality trends in the majority of other age groups (*P* < 0.05).

**Table 2:** Joinpoint regression analysis of pancreatic cancer mortality in Serbia, excluding the Autonomous Province of Kosovo and Metohia, by sex and age, from 1991-2010

Average age-specific rate		
			Age*	rate (per 100 000)	average annual percent change (95% confidence interval [CI])
Men		
40-44	3.15	+ 0.4 (-3.1 to 4.0)
45-49	6.26	+ 0.9 (-0.9 to 2.8)
50-54	13.03	- 0.3 (-1.3 to 0.8)
55-59	23.36	+ 2.6^‡^ (1.4 to 3.8)
60-64	30.39	+ 2.1^‡^ (0.7 to 3.4)
65-69^†^	43.00	+ 2.6^‡^ (1.1 to 4.2)
70-74	55.71	+ 1.0 (-0.1 to 2.2)
75-79	60.62	+ 1.6^‡^ (0.7 to 2.6)
80-84	72.15	+ 1.0 (-1.2 to 3.1)
85+	55.37	+ 2.6^‡^ (0.6 to 4.7)
All men		+ 1.6^‡^ (1.1 to 2.0)
Women		
40-44	1.51	- 2.0 (-6.1 to 2.3)
45-49	3.34	+ 1.0 (-2.0 to 4.0)
50-54	6.85	+ 2.9^‡^ (0.8 to 5.0)
55-59	11.22	+ 2.4^‡^ (0.9 to 3.8)
60-64	18.95	+ 1.9^‡^ (0.8 to 3.0)
65-69	26.43	+ 1.5^‡^ (0.4 to 2.5)
70-74	37.39	+ 2.5^‡^ (1.7 to 3.3)
75-79^§^	46.07	+ 3.0^‡^ (1.0 to 5.0)
80-84	51.88	+ 3.1^‡^ (1.4 to 4.8)
85+	44.83	+ 4.3^‡^ (2.6 to 5.9)
All women		+ 2.2^‡^ (1.7 to 2.7)

*Joinpoint results are not shown for the age subgroups <40, because there were fewer than 5 cases in any year.

†Trend 1 (1991-2004): annual percent change (APC) (95% CI) = +0.9 (-0.4 to 2.2); Trend 2 (2004-2010): APC (95% CI) = +6.6^‡^ (2.2 to 11.2).

‡Joinpoint is significantly different from zero at alpha = 0.05.

§Trend 1 (1991-2000): APC (95% CI) = -0.3 (-3.4 to 3.0); Trend 2 (2000-2010): APC (95% CI) = 5.9^‡^ (3.1 to 8.9).

## DISCUSSION

This study described pancreatic cancer mortality trends for Serbian population in the last two decades and found an increasing trend in both sexes and most age groups. Age-standardized pancreatic cancer mortality in Serbian men of 8.29 per 100 000 in 2008 is high compared with other European countries (3). The highest mortality rate in men was reported in Hungary (11.56 per 100 000), followed by Armenia (10.81), Albania (10.70), Croatia (9.47), and Russian Federation (8.83). The highest mortality rate in women was found in Finland (7.14 per 100 000), Czech Republic (7.13), and Hungary (7.07). The lowest mortality rates were found in Cyprus and Turkey, both in men (3.69 and 3.72 per 100 000, respectively) and women (2.43 and 2.49 per 100 000, respectively). Rates for both sexes were higher in eastern Europe than in the European Union (18,19). In Serbian population, as well as in other populations (3), pancreatic cancer deaths rates were higher in men than in women.

Worldwide trends for both sexes in the second half of the 20th century varied considerably (6,19). They were increasing throughout Europe (the European Union and 6 selected eastern European countries) between the late 1950s and the 1980s, and leveled off in the 1990s (19). In the USA, they steeply increased until the mid-1970, when they reached a peak, then gradually declined by 0.1% per year until 2001, after which they began to rise until 2009 (by 0.5%) (6,20).

Pancreatic cancer mortality rates in Serbia showed an increasing trend in both sexes. An increasing trend in both sexes was also noticed in most countries of central, eastern, and southern Europe (8,21). A decreasing trend, on the other hand, was found in men in Denmark, the Netherlands, the United Kingdom, and in Nordic countries, and a level-off was observed in France, Portugal, and Austria (8,22). Yearly mortality rates from 2002 to 2009 have been slightly rising in the USA, by 0.5% in men and by 0.4% in women (6). Also in Japan (23), at the end of the 1990s, pancreatic cancer mortality increased by 0.4% per year in women aged 65-84, and decreased by 0.2% in men of the same age. Recent pancreatic mortality trends in the Netherlands, Norway, Poland, and Switzerland in the age group 35-64 years were favorable for men, but unfavorable for women (8).

Since diagnostic and treatment improvements in the last decades (24,25) have not substantially influenced the survival rate of pancreatic cancer, its mortality rate still depends mainly on its incidence. International differences in mortality rates and temporal trends suggest that etiology of pancreatic cancer is influenced by environmental factors, especially smoking, but also by nutritional and dietary factors, obesity, alcohol use, and diabetes mellitus (26-29).

The strongest environmental risk factor for pancreatic cancer is smoking. For example, the relative risk of pancreatic cancer in smokers compared to non-smokers was 2.0 (29,30) and the proportion of pancreatic cancer attributable to cigarette smoking in US African-Americans was 29% and 26% in Caucasians (31). As for other tobacco-related malignant tumors, international disparities in mortality trends most likely reflect different rates of tobacco use (30-34). Tobacco exposure in Serbia is high. The percentage of male and female smokers in Belgrade in 1976 and 1977 was 49% and 25%, respectively; in 1988 and 1989, 51% and 37% (35), and in 2000, 48% and 38% (32). The antismoking campaign, which was intensified from 2000, contributed to a decrease in the number of smokers (38% of men and 30% of women in 2006) (36). Serbian joining of the World Health Organization Convention on Tobacco Control in June 2006 is expected to bring about a further reduction in the number of smokers.

However, there are also other important risk factors for pancreatic cancer; according to the data for 2006, 3.4% of the adult population of Serbia consumed alcohol on a daily basis, which is an increase of 0.1% compared with 2000 (3.3%) (36). As in many other countries, the frequency of obesity and diabetes in Serbia has been increasing, and the increase is estimated to continue (37,38). In 2000 as in 2006, one in two adults was overweight, 18.3% were obese, and 36.2% were pre-obese, and the number of overweight and obese 7-19 years old children increased from 12.6% to 18.0% (36). In 2006, about 6.8% of adults in Serbia had diabetes mellitus (6.2% of men and 7.5% of women), which is significantly higher than 4.8% in 2000 (4.5% of men and 5.0% of women) (39,40). Taking all this into account, it seems very difficult to predict the future pancreatic cancer mortality trend, although moderate and insignificant mortality changes in younger age groups are promising.

A limitation of the present study is related to the reliability and validity of death certificates. The World Health Organization assessed the quality of Serbian data on the cause of death as moderate (41). Besides that, pancreatic cancer is difficult to diagnose and the increasing mortality trends may be related to greater diagnostic accuracy (20). It can be assumed that diagnostics of pancreatic cancer was worse during the first half of the observed period (from 1991 to 2000) than during the second half, because of the war and economic crisis in the country. The proportion of cases with uncertain death cause (revision 9 codes 780-799 and revision 10 codes R00-R99) in the observed period was on average 6.8%, with a non-significant decreasing trend (*P* = 0.137). In the majority of developed countries, the quality of death certification is better than in Serbia. For example, the proportion of uncertain causes of death in Nordic countries is 1% (42) and 5% in China (43). We have no data on changes in the autopsy rate during the studied period, which could influence the mortality coding. Registration of autopsies in Serbia began in 2006. From 2006 to 2010, autopsy was performed in 2.1% of all deaths and 2.6% of pancreatic cancers diagnosed at autopsy were missing on the death certificate. We also have no data on possible changes in survival of pancreatic cancer patients, but it is not probable that survival deteriorated during the observed period since the treatment progress has been negligible (44-46). During the study period, there was a change from ICD9 to ICD10, but it seems not to have had an effect on the overall rates of pancreatic cancer. There are no data on pancreatic cancer incidence for the entire Serbia without Kosovo and Metohia (Central Serbia and Vojvodina). For the period 1999 to 2010, there are only data for Central Serbia, showing a non-significant increasing trend of age-standardized pancreatic cancer incidence in both sexes (*P* = 0.338 in men and *P* = 0.679 in women) (47).

Serbia is among the countries with the highest pancreatic cancer mortality in the world. An increasing mortality trend was present in both sexes and in most age groups. Pancreatic cancer is one of the leading causes of cancer death in Serbia and therefore further effort should be made to clarify its etiology and risk factors.
